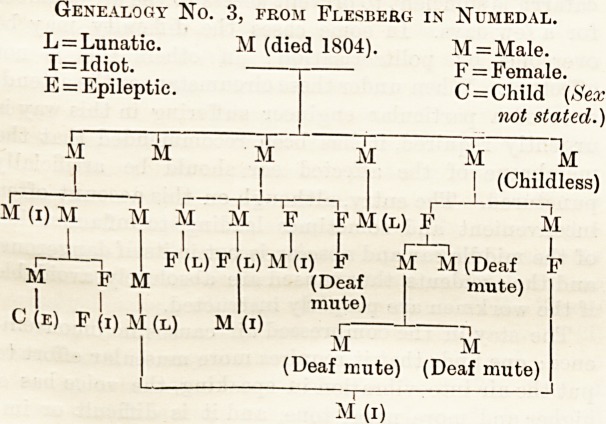# Hereditary Neuroses in Children.—II

**Published:** 1897-05-22

**Authors:** 


### Modern Sociology.

### HEREDITARY NEUROSES IN CHILDREN.—II.

We cannot more forcibly illustrate the statements put forward in our preceding article than by transcribing one of Dahl's pedigrees (translated in Dr. Ireland's book on Idiocy).

Genealogy No. 3, from Flesberg in Numedal.

It would appear from the above that in the three generations in which neuroses appeared, consisting of 27 persons, there were five idiots, four lunatics, four deaf and dumb, and one epileptic. Dahl discovered, by comparing the history of this family with that of others in the same parish, that the tendency to neurosis was fifteen times as great in the former as in the latter.

As we have previously seen, a degenerating family tends to pass from the stage of irregular or disorderly nervous and mental action through that of mental imperfection to extinction. The neurosis affecting the parent is apt to express itself in some abnormality in the offspring. It may affect the original formative process which occurs in uterine gestation. It would seem that in some cases a lack of vigour in the male element, in others trophic imperfections due to maternal neuroses, may be determining causes of defects of embryonic development. We may instance paternal senility, or the constitutional taint of syphilis or of phthisis, as examples of the former: debilitating disease or nervous shock affecting the mother during pregnancy, as examples of the latter. There is no doubt that malformations are met with amongst children of neurotic parents in greater proportion than amongst others. Instances are frequent amongst such of malformations of fingers and toes, of hare-lip, of cleft palate or mis-shapen palate, of defects of the organs of the senses (*e.g.*, congenital deafness or blindness), and of physical as well as functional imperfections of the higher nerve-centres. Under the last category we may class congenital idiocy and imbecility, and, according to the statistics of Dr. Fletcher Beach with regard to 1,180 patients at the Darenth Asylum, no less than 22·71 of the idiots there had a family history of insanity, and 36·86 per cent. of epilepsy or some other neurosis.* The same authority gives 19·57 per cent. as the proportion in which a parental tendency to alcoholism existed, (Dr. Shuttleworth stating his percentage at 13·25). It is not, however, in gross defect alone that inherited neurosis expresses itself; frequently, indeed, its influence is more subtle, and its impress affects the ultimate constitution of the nerve elements themselves. In such cases these appear to have an inherent instability of composition (to borrow a chemical phrase), with resulting tendency to explosive action, which differentiates them from the normal nerve cell in spite of resemblance of anatomical structure. The difference would seem to be dynamic, and children born with this peculiarity of nervous constitution are prone to break down under the strain of developmental changes through which others safely pass. There may be no abnormality evident at birth; but the baby is soon recognised as a "tiresome child," sleeping badly and often a "screamer." The period of dentition is apt to lead to a crisis, reflected alveolar irritation giving rise to convulsive symptoms. Teething-fits may indeed, in the majority of instances, be looked upon as a manifestation of hereditary neurosis, and although (as Henoch* has pointed out) they are most common in rickety children, it is probable, as Dr. Clouston says, that both rickets and convulsions may be attributed to a common cause, viz., atrophic neurosis.† Teething-fits are apt to be assigned by parents as the origin of the mental defect of their offspring; and in the article in Hack Tuke's Dictionary previously referred to they figure as a factor in 32·58 per cent. of Dr. Shuttleworth's cases, and in 22·11 of Dr. Beach's. But in truth they are not often the original cause; in a large proportion of the cases a neurotic family history had also been ascertained, and it may be safely asserted as a rule that where teething fits eventuate in mental deterioration some hereditary neurosis is present. Hydrocephalus is sometimes associated with eclampsia, more especially in rickety cases, and may be looked on as another form of hereditary neurosis.

Epilepsy, as distinguished from eclampsia (with which, however, it may be connected), manifests itself, according to Gowers, during the first seven years of life, in the case of 23 per cent. of epileptics; and as many as 5 per cent. of the first attacks arise during the first year of life. A strong neurotic heredity marks these latter; and a family history of epilepsy or insanity was traced by Gowers in one-third of his 1,450 cases. Beach ascertained the existence of a family history of epilepsy in 14·06 per cent. of his 1,180 imbecile patients. A typical case of developmental epilepsy is cited by Clouston,‡ in which the first fit came on at the age of fourteen in a boy previously healthy, but whose paternal grandfather was drunken and insane, paternal grandmother epileptic, father highly neurotic, and the mother alcoholic.

Nervous headache in childhood is often the sign of neurotic inheritance. Sometimes it takes the form of migraine, and Henoch refers to the case of a child of two and a half years who showed decided symptoms of this affection. He remarks on the frequent occurrence of nervous heredity in such cases; and in the other direction it is worthy of note that the late Dr. Kerlin found a family history of migraine in 5 per cent. of the feeble-minded cases,§ which he tabulated etiologically.

* Hack Tuke's "Dictionary of Psychological Medicine," Vol. II., p. 664; "Etiology of Idiocy," Beach and Shuttleworth.

* "Lectures on Children's Diseases," Vol. I., p. 166.
† "Neuroses of Development," p. 61.
‡ "Neuroses of Development," p. 105.
§ Transactions of Association of Medical Officers of American Institutions for Feeble-minded, &c., 1880, p. 150, *seq.*

## Figures and Tables

**Figure f1:**